# Optimization of pulmonary blood flow analysis method using dynamic chest radiography

**DOI:** 10.1002/acm2.70588

**Published:** 2026-04-15

**Authors:** Tasuku Nakajima, Ryuichi Ushijima, Rie Tanaka

**Affiliations:** ^1^ Department of Radiology Toyama University Hospital Toyama Toyama Japan; ^2^ Division of Health Science Graduate School of Medical Sciences Kanazawa University Kanazawa Ishikawa Japan; ^3^ Second Department of Internal Medicine University of Toyama Toyama Toyama Japan; ^4^ College of Medical, Pharmaceutical & Health Sciences Kanazawa University Kanazawa Ishikawa Japan

**Keywords:** chronic thromboembolic pulmonary hypertension, dynamic chest radiography, flat panel detector, pulmonary blood flow analysis

## Abstract

**Background:**

Pulmonary blood flow analysis was conducted utilizing dynamic chest radiography (DCR) and pixel values assessed within a region of interest (ROI) in the left ventricle as reference signals. However, the effect of the ROI placement on the analysis accuracy is still uncertain.

**Purpose:**

This study aimed to identify the ROI locations that produced clinically useful images with higher signal values.

**Method:**

A total of 40 DCR, single‐photon emission computed tomography, computed tomography angiography (CTA), and angiography image data from 15 patients with chronic thromboembolic pulmonary hypertension were analyzed. The DCR images were analyzed, and the time difference rate of each peak value (PD%) was calculated based on the correlation between the reference signal and heart rate phase. To clarify the ROI for acquiring clinically valuable high‐signal‐value images, the ROI locations of the high‐ and low‐signal‐value images were categorized, and PD% values were statistically compared across the ROI locations. Furthermore, the ROI locations of the high‐ and low‐signal‐value images were visually inspected for anatomical positions using the CTA images.

**Results:**

There were significant differences in PD% between high‐ and low‐signal‐ value images when the ROIs were positioned within the upper and inside heart regions on DCR (median PD%: 4.48% and 4.41%, respectively; *p* < 0.05). The ROI location of the low‐signal‐value images on CTA tended to be in the middle of the heart, encompassing the right ventricular wall and the interior, excluding the left ventricular cavity.

**Conclusions:**

The study identified the ROIs that could yield clinically valuable images with higher signal values in the DCR‐based analysis of pulmonary blood flow. This discovery is anticipated to enhance the efficiency and reliability of pulmonary blood flow imaging for diagnosing chronic thromboembolic pulmonary hypertension with DCR.

## 1 INTRODUCTION

Chronic thromboembolic pulmonary hypertension (CTEPH) is a condition where a thrombus or embolus in the pulmonary artery persists despite appropriate coagulation therapy, leading to abnormally elevated pulmonary artery pressure.^1^ International epidemiological analysis has projected an increase in the global patient population between 2015 and 2025.^2^ The etiology of this disease remains unknown and is under investigation worldwide.^3^ The definitive diagnosis of CTEPH relies on a comprehensive review of various imaging findings, making diagnostic imaging crucial in daily clinical practice.^4^ Currently, pulmonary blood flow scintigraphy, pulmonary arteriography, and contrast chest computed tomography (CT) are used to diagnose CTEPH. They have been reported to be useful for evaluating blood flow defects and thrombi. However, these procedures are invasive due to punctures and the use of radioisotope drugs or contrast media.^5^ Therefore, we focused on minimally invasive, cost‐effective dynamic chest radiography (DCR) and video analysis technology for hemodynamic evaluation. DCR, a functional x‐ray imaging technique utilizing a flat‐panel detector (FPD) and pulsed x‐ray generator, continuously captures breathing or breath‐holding conditions.^6^ In commercially available DCR‐based pulmonary blood flow analysis, pixel values (∝ FPD Achievable dose) measured in a region of interest (ROI) automatically located in the left ventricle serve as a reference signal to estimate heart rate phase as a substitute for electrocardiogram. The temporal correlation with each pixel value in the lung region is visualized as a color‐mapping image (referred to as pulmonary blood flow images).^7^, ^8^, ^9^, ^10^ Previous studies have shown that DCR‐based pulmonary blood flow imaging in patients with CTEPH aligns with the imaging findings of pulmonary blood flow scintigraphy, computed tomography angiography (CTA), and pulmonary angiography.^11^ In addition, DCR demonstrated good correlation with pulmonary blood flow scintigraphy in subsequent evaluations.^12^ Despite several studies exploring the potential of DCR for quantifying pulmonary circulation, large‐scale validation studies comparing DCR with established gold‐standard modalities remain insufficient. Consequently, qualitative visual assessments remain prevalent in clinical applications. Technical challenges persist, including the need to refine computational and processing analyses and establish normative reference values. Notably, the optimal anatomical location of the reference ROI within the analysis workflow lacks standardization.^13^ Furthermore, if the reference signal is not properly obtained, the acquisition and visualization of the blood flow signal may be compromised, potentially resulting in false positives. Therefore, establishing a logic for setting the reference ROI location is crucial. The reference ROI location is automatically determined based on ventricular wall motion using motion‐tracking techniques to generate a pulmonary blood flow image.^6^ Incorrect ROI placement necessitates manual replacement for reanalysis, a time‐consuming and complex process. Once the logic for setting the reference ROI location is established, it is anticipated to facilitate efficient manual correction of the ROI location and ensure stable pulmonary blood flow signal values. This study aimed to identify an ROI that can yield clinically valuable high‐signal blood flow images for pulmonary blood flow analysis using DCR.

## 2 METHODS

### 2.1 Subject

This retrospective study included all patients admitted within the approved timeframe by the institutional ethics review board. To ensure diagnostic accuracy for CTEPH, only patients with a definitive diagnosis established through imaging modalities and clinical evaluations were included. The study collected a total of 40 DCR, single‐photon emission computed tomography (SPECT), CTA, angiography (Angio) image data from 15 patients with CTEPH (aged 32–90 years; median, 73.6 ± 14.36 years; M:F = 4:11; pulmonary artery pressure, 34.87 ± 9.13 mm Hg; WHO functional class II:III:IV = 3:11:1) who visited the Department of Cardiology at Toyama University Hospital between 2020 and 2023. However, five cases (two patients) where the left ventricle was completely obscured by the right ventricle in the frontal view on the CTA images were excluded. Consequently, 35 patients (DCR, 35; SPECT, 15; CTA, 13; and angiography, 34) were included in the visual evaluation and signal value analysis. For the anatomical evaluation of the ROI location, 15 cases with DCR and CTA performed within 3 months were included due to changes in right ventricular hypertrophy in CTEPH with treatment status. This study received approval from the University of Toyama Ethical Review Committee for Clinical and Epidemiological Research (approval number R2022196).

### 2.2 Image acquisition

All patients underwent DCR with a 7‐second inspiratory breath‐hold using a dynamic digital radiography system comprising an FPD (AeroDR fine, Konica Minolta, Tokyo, Japan) and a pulsed x‐ray generator (Radspeed Pro, Shimadzu Corporation, Kyoto, Japan). Imaging was conducted in the standing position in the posteroanterior direction at a rate of 15 frames per second. The imaging parameters were as follows: 100 kV, 0.16 mAs per pulse, source‐to‐image distance of 180 cm, field of view measuring 43 cm × 43 cm, a matrix size of 1024 × 1024 pixels, pixel dimensions of 417 × 417 µm^2^, and a 16‐bit grayscale image range. SPECT scans were acquired using a SPECT/CT scanner (Symbia T2 SPECT/CT; Siemens Healthcare, Forchheim, Germany). Images were captured 5 min after the administration of 260 MBq of ^99m^Tc‐MMA and the injection of 10 mL of saline solution. CTA was performed using a 192‐slice CT scanner (SOMATOM Force; Siemens Healthcare, Forchheim, Germany). The scanning parameters used in the protocol were as follows: tube voltage of dual‐energy Sn150 kV/90 kV and a reference tube current–time product of 310 mAs. The contrast medium injection protocol involved iopromide at a concentration of 550 mg/mL, an injection time of 25 s, and a saline solution injection volume of 15 mL. Angio was conducted using an automatic contrast medium injection system (Avanta Arterion, Bayer, Nordrhein, Germany) or a manual injection method, with digital subtraction angiography (DSA) or digital angiography (DA) utilized as the imaging techniques. The contrast media injection protocols were as follows: DSA (injection speed of 7 m/s, injection volume of 25 mL) and DA (manual injection volume of 5–10 mL) using a nonionic contrast medium (iopamidol; 370 mgI).

### 2.3 Time difference rate of peak pixel value (PD%)

**FIGURE 1:**
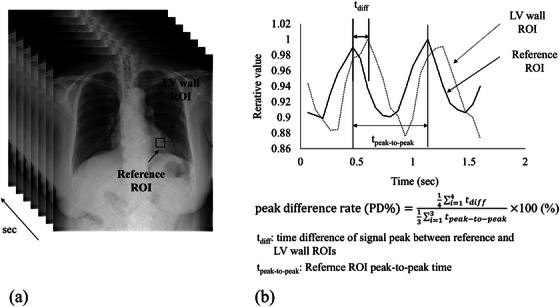
Peak difference rate (PD%). (a) ROI of the left ventricle wall and reference ROI location on dynamic chest radiographs. The squares in the figure indicate the ROI locations. (b) Calculation of PD%.

Pulmonary blood flow images were acquired from sequential DCR images using a workstation (KINOSIS; Konica Minolta, Tokyo, Japan). The analysis method for pulmonary blood flow is based on correlating a reference signal from the left ventricle with that from the lung fields, where a strong correlation appears as a red‐colored area.^6^, ^14^, ^15^ The pulmonary blood flow images were generated utilizing a reference signal measured in ROIs automatically placed on the ventricular wall (referred to as Auto image) and those measured in manually adjusted ROIs (referred to as Manual image). A cardiologist with 17 years of experience and a radiologic technologist with 16 years of experience analyzed the correlation between the reference signal and heart rate phase using ImageJ software (Version 1.53t, National Institutes of Health, Maryland, USA). An ROI of 1.5×1.5 cm was manually positioned at the same location as the automatic and manual ROIs on the KINOSIS workstation, and the temporal changes in average pixel values within the ROI were recorded. If the location of ROI differed, the observers discussed the location to reach a consensus. In addition, the original DCR images were examined to extract the signal waveform from the ROI on the left ventricular wall representing the heart rate phase, along with the signal waveform from the reference ROI. Subsequently, the time difference of each peak value (PD%) was calculated as the average of the four waveforms (Figure 1). Typically, three heart rate cycles are adequate for accurate temporal assessment of the heart rate phase.^16^ However, to determine the peak‐to‐peak time of the reference ROI four waveforms are necessary to obtain information from three cardiac cycles. Therefore, the calculations in this study were based on the analysis of the four waveforms.

### 2.4 Visual evaluation of clinically useful high‐signal‐value images

**FIGURE 2:**
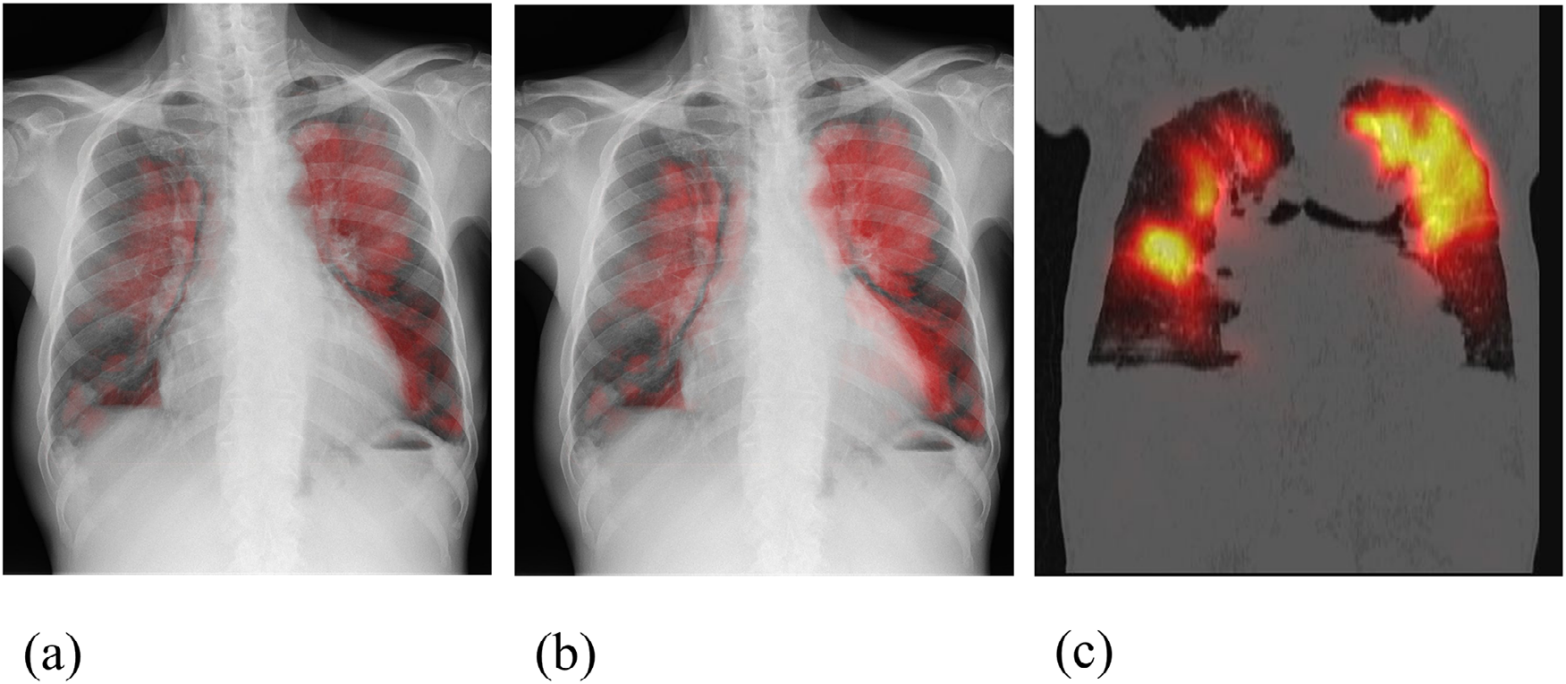
A visual evaluation of the normal blood flow area in a 76‐year‐old male diagnosed with chronic thromboembolic pulmonary hypertension. (a) Maximum intensity projection of low‐signal‐value images. (b) Maximum intensity projection of high‐signal‐value images. (c) Single photon emission computed tomography images used as gold standard.

The normal blood flow area was comprehensively determined based on a diagnostic workflow that integrated SPECT, CTA, and Angio images and was used as the gold standard (GS). To elucidate the trend of PD% in clinically useful pulmonary blood flow images, the visual similarity between the GS and the normal blood flow area identified in both the Auto and Manual pulmonary blood flow images was visually assessed in 35 cases. An image displaying a higher blood flow signal value in the normal blood flow areas identified on the GS was categorized as a high‐signal‐value image, while an image showing lower blood flow signal values in the normal blood flow areas identified on the GS was classified as a low‐signal‐value image. In the absence of significant difference between Auto and Manual images, both images were considered high‐signal‐value images. For ease of visual assessment, maximum intensity projection (MIP) images of high‐ and low‐signal‐value images were generated in the time‐axis direction using free image analysis software. Figure 2 illustrates examples of high‐ and low‐signal‐value images utilized as GS and MIP images, respectively.

### 2.5 Comparison of PD% between auto and manual images

To elucidate the comparison between automatic and manual analysis of clinically relevant high pulmonary blood flow images, the visual resemblance between the GS and the normal blood flow region identified in both Auto and Manual pulmonary blood flow images was visually evaluated in 35 cases and categorized into three groups. The group with only auto images considered to have a high‐signal‐value was classified as Auto image‐superiority; the group with both Auto and Manual images considered to have a high‐signal‐value was classified as having no difference in image quality; and the group with only Manual images considered to have a high‐signal‐value was classified as Manual image‐superiority. For each group, the PD% was compared between automatic and manual images to identify the trend of change in PD% by Manual manipulation.

### 2.6 Anatomical sites evaluation of ROI location

**FIGURE 3:**
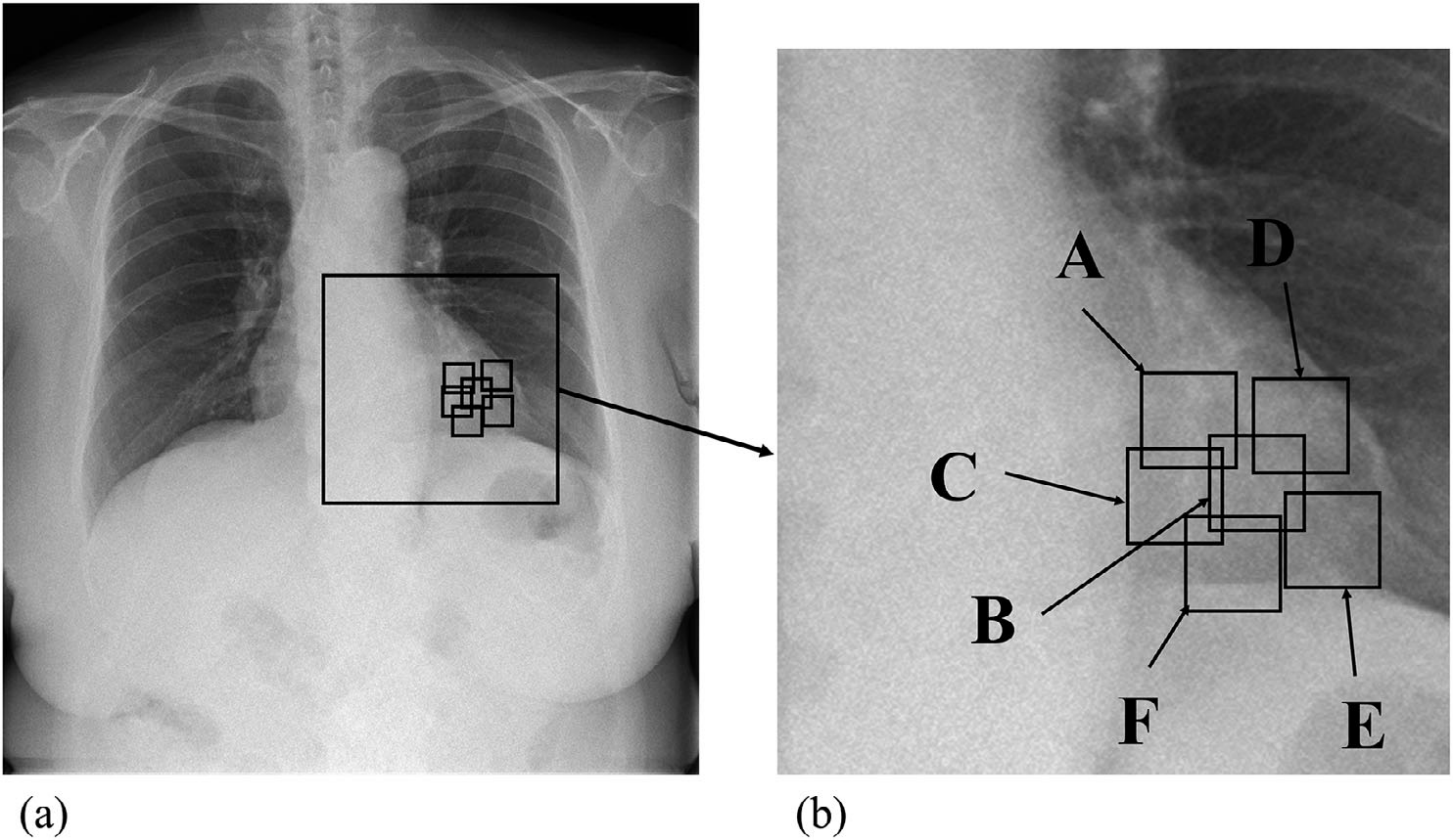
Location of ROIs. (a) One representative frame in DCR. The squares in the figure indicate the location of the magnified view. (b) An enlarged view of the heart area on the DCR image. The squares in the figure indicate ROIs located around (A) the left ventricular outflow tract upper, (B) the middle of the left ventricle, (C) the inside of the left ventricle, (D) the outside of the left ventricle, (E) the left ventricular apex, and (F) the lower of the left ventricle.

To clarify the ROI locations for obtaining high signal value images, the ROI locations on the DCR images were categorized into six groups for both high‐ and low‐signal‐value images: outside, middle, inside, upper, apex, and lower (Figure 3). Additionally, the ROI locations of the 30 datasets (15 high‐ and 15 low‐signal‐value images) were visually evaluated for the position and anatomical overlap of the left ventricular lumen, right ventricular wall, and left atrium in the 3D images generated from the CTA images. All assessments were visually conducted by a cardiologist with 17 years of experience and a radiologic technologist with 16 years of experience; any discrepancies were resolved through consensus.

### 2.7 Statistical analysis

Statistical analysis of PD% was performed using statistical analysis software (SPSS version 26, IBM, Chicago, USA). The distribution of PD% was evaluated using the Shapiro–Wilk test, which revealed a departure from normality (*p* < 0.05). Consequently, a non‐parametric statistical approach was employed to analyze the PD%. The Wilcoxon test was utilized to assess the significance of the difference in PD% between high‐ and low‐signal value images (*p* < 0.05). Similarly, the Wilcoxon test was applied to examine the statistical variance in PD% between Auto images and Manual images across the categories of auto image superiority, no difference, and Manual image superiority (*p* < 0.05). Furthermore, the Kruskal–Wallis test was employed to investigate potential variations in PD% based on ROI location (*p* < 0.05), with subsequent Wilcoxon tests conducted for multiple comparisons (*p* < 0.05).

## 3 RESULTS

### 3.1 Visual evaluation and comparison of PD% between high and low signal value images

Pulmonary blood flow images in all 35 cases were visually similar to those of the GS. Out of the total 70 images, comprising 35 Auto and 35 Manual images, 45 were categorized as high‐signal values, while the remaining 25 were classified as low‐signal values.

The median and interquartile range (IQR) of PD% for the high‐signal‐value image group were 5.56% (2.78–8.82), while for the low‐signal‐ value image group, they were 9.00% (5.00–13.82). The PD% values were significantly lower in the high‐signal‐value images compared to the low‐signal‐value images (*p* < 0.05).

### 3.2 Comparison of PD% between auto and manual images

**FIGURE 4:**
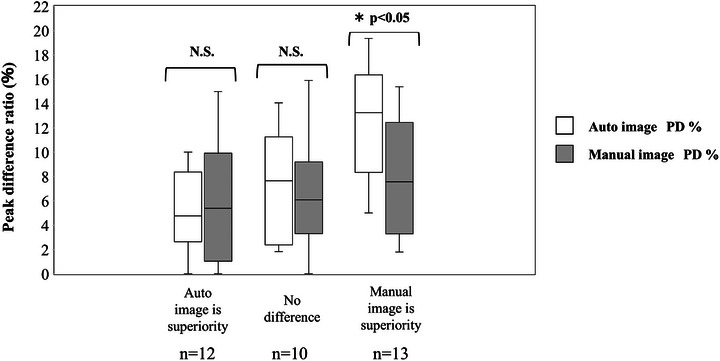
Box‐and‐whisker plot illustrating the PD% between Auto and Manual images categorized as Auto image superiority, no significant difference in images, and Manual image superiority. The upper and lower error bars represent the maximum and minimum values, respectively. The comparison showed no statistical significance (N.S.). *p < 0.05.

Figure 4 shows a box‐and‐whisker plot illustrating the PD% values for Auto and Manual images, categorized as Auto image‐superiority, no significant difference in images, and Manual image‐superiority. The results, encompassing classification counts derived from visual assessment (12 Auto image‐superiority, 10 no significant difference, and 13 Manual image‐superiority) and comparative analysis, are consolidated in Figure 4. The median and IQR of PD% in Auto and Manual images were 4.07% (2.63–8.73) and 5.38% (1.04–9.92) for the Auto image superiority group, 7.65% (2.38–11.26) and 6.09% (3.31–9.21) for the no difference in image group, and 13.26% (8.35‐16.36) and 7.56% (3.29‐12.45) for the Manual image superiority group, respectively. There was no significant difference in PD% between Auto image and Manual image for the Auto image superiority group and the no‐difference group (*p* > 0.05), while the PD% of Manul image was significantly lower than that of Auto images (*p* < 0.05) for the Manul image superiority group.

### 3.3 Comparison of PD% by ROI location on DCR images

Table 1 shows the number of high‐signal and low‐signal‐value images, along with the mean PD% and IQR. The results of the Kruskal–Wallis test are detailed in Table 1. The high‐signal‐value images were most prevalent in the upper region, followed by the middle, inside, outside, and equal numbers at the apex and lower regions. The median PD% ranked from smallest to largest as follows: upper, inside, middle, lower, apex, and outside. Although the count of high‐signal‐value images rose with the ROI positioned in the middle or inside, the count of low‐signal‐value images also increased. Moreover, the number of low‐signal‐value images surpassed that of high‐signal‐value images when the ROI was situated outside. The Kruskal–Wallis test revealed a significant disparity in PD% between the high‐ and low‐signal‐value images (*p* < 0.05). The Wilcoxon test for PD% exhibited notable distinctions for inside‐outside, upper‐outside, and middle‐outside (*p* < 0.05), whereas no significant variations in PD% were observed for the other ROI location combinations (*p* > 0.05).

**TABLE 1:** Number of high‐signal‐value and low‐ signal‐ value images, median PD% in each ROI location.

ROI location (Number of images)	High‐signal‐value images	Low‐signal‐value images	PD% median (IQR)	*p* value
Upper (*n* = 16)	16	0	4.41(0.00–5.56)	
Middle (*n* = 23)	14	9	7.63(4.92–8.74)	
Inside (*n* = 13)	8	5	4.48(2.50–8.44)	^＊^0.03
Outside (*n* = 10)	4	6	13.54(10.09–11.72)	
Apex (*n* = 3)	2	1	15.29(15.19–15.31)	
Lower (*n* = 5)	2	3	5.38(3.00–4.41)	

Parentheses indicate IQR.

^＊^ *p* < 0.05.

Table 2 displays the positions of the ROIs before and after manual adjustment for manual image enhancement (*n* = 13). The count of ROIs post manual correction decreased in the sequence: upper > inside > lower > middle = apex. Regarding Manual images‐superiority, when the ROI location before manual correction was in the middle or inside, the ROI location after manual correction was often on the upper side. However, when the ROI locations before manual correction are outside, the ROI locations after manual correction are often inside

**TABLE 2:** Number of ROI locations before and after manual corrections in Manual images superiority (*n* = 13).

		After manual corrections				
Before manual corrections		Upper	Middle	Inside	Apex	Lower
Location of automatically set ROI	Middle	3	‐	0	1	2
	Inside	2	0	‐	0	0
	Outside	0	1	3	0	0
	Lower	1	0	0	0	‐
Total		6	1	3	1	2

### 3.4 ROI location on CTA images corresponding to clinically useful high‐signal‐value images

**FIGURE 5:**
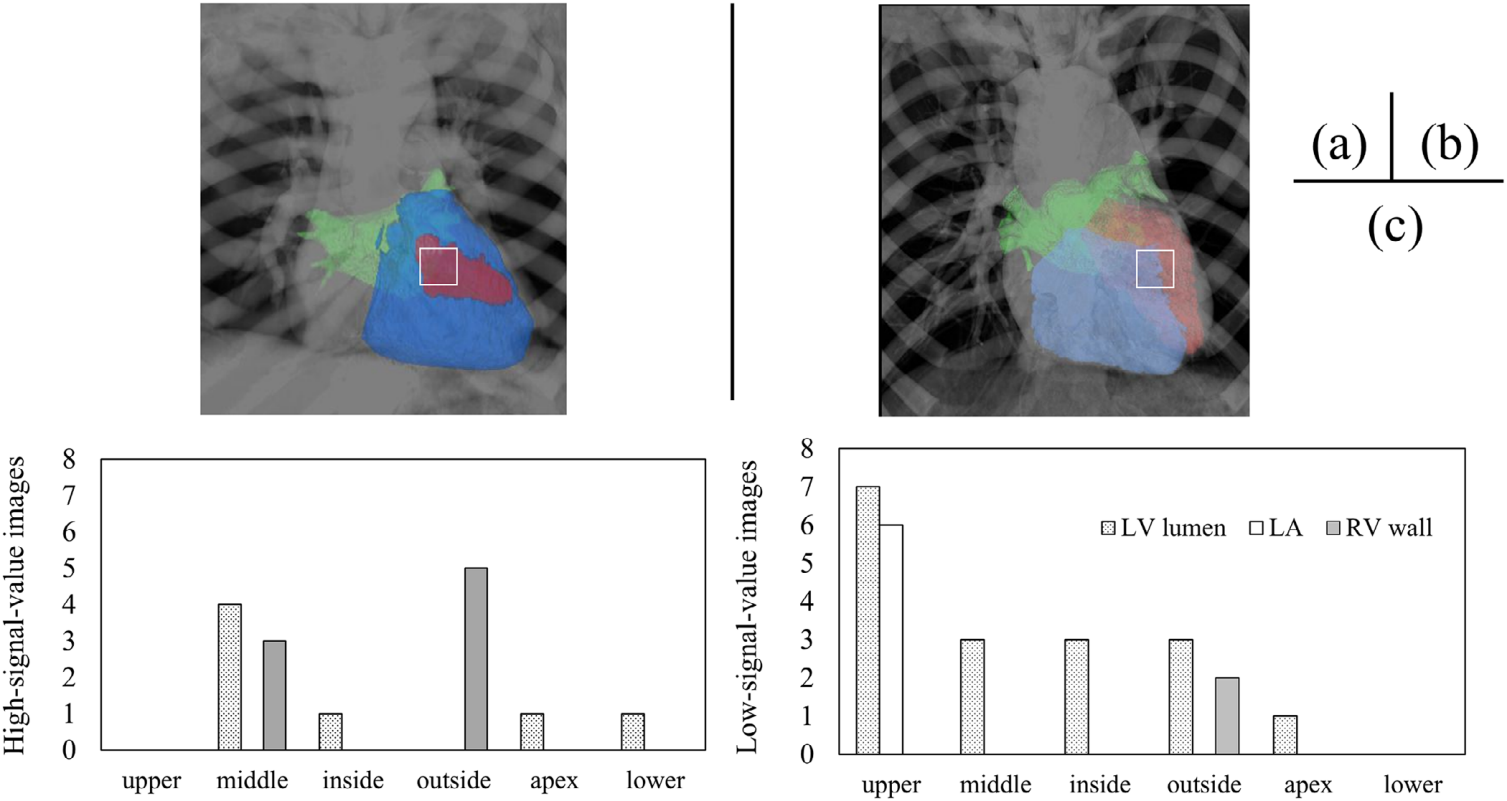
Results of an anatomical analysis of the ROI location using CTA images. (a) One example with ROI capturing the left ventricular lumen and left atrium. (b) One example with ROI capturing the ventricular lumen and right ventricular wall. (c) Number of cases in which the left ventricle lumen (LV), the left atrium (LA), and the right ventricular (RV) wall were captured in each ROI location. The squares in the figure depict the location of ROI. The red, blue, and green areas correspond to the left ventricular lumen, the right ventricle, and the left atrium, respectively.

The ROI location of the high‐signal‐value image was on the left ventricular lumen in the CTA images, and the ROI avoided the right ventricular wall in 15 of the 17 images. In contrast, the ROI location of the low‐signal‐value images did not capture the left ventricular lumen and captured the right ventricular wall in 9 of the 13 images. The ROI was located in the upper area that captured the left atrium in six of the seven images, but high‐signal‐value images were provided in all images. In addition, the ROI location was outside, which captured the right ventricular wall in seven of eight images and low‐signal value images in five images. In cases where the ROI location was in the middle, it captured the right ventricular wall in three out of seven images and low signal value images in all three cases (Figure 5).

## 4 DISCUSSION

Manual manipulation of the ROI location was effective in 13 of the 35 images, resulting in high‐signal value‐images with a significantly lower PD%. When using DCR images, there were significant differences in PD% between high‐ and low‐signal‐value images when the ROIs were located in the inside and upper heart regions (*p* < 0.05). In contrast, when using CTA images, the ROI location of low‐signal‐value images tended to be in the middle of the heart, capturing the right ventricular wall and the inside, excluding the left ventricular cavity. These results suggest that manual correction of the ROI location to avoid the right ventricular wall and include the left ventricular cavity can reduce the PD% and produce high‐signal‐value blood flow images in DCR. The manual image superiority group accounted for approximately 37% of all images, and higher blood flow signal values were obtained in pulmonary blood flow images when the PD% value was smaller. The pixel value of the ROI location on the left ventricular wall reached its maximum value during the late diastole to early systole phases (R wave of the electrocardiogram). These results suggest that higher blood flow signal values can be obtained from pulmonary blood flow images by placing the reference ROI in a position where the left ventricle motion can be accurately monitored. By contrast, when the ROI captured the right ventricular wall, lower blood flow signal values were obtained in the pulmonary blood flow images. This can be explained by the fact that patients with CTEPH have right ventricular enlargement due to right heart failure, where the right ventricular wall is more likely to be captured when the reference ROI is located outside or in the middle.^17^, ^18^, ^19^ Heart failure with pulmonary hypertension, as typified by CTEPH, may also be associated with a larger time difference between right and left ventricular wall motion.^20^ In addition, the reference ROI did not capture the left ventricular cavity or lower blood flow signal value in the pulmonary blood flow images, suggesting that the reference ROI captured the movement of different organs and blood flow, respectively. If the pixel values in the reference ROI do not accurately monitor the motion of the left ventricle, the blood flow signal value in the pulmonary blood flow images will be lower. These results suggest that the most accurate reference ROI for monitoring left ventricular motion is the location that avoids the right ventricular wall and captures the inside of the left ventricular cavity, thus providing images with high blood flow signal values for pulmonary blood flow. However, locating the left ventricular lumen and right ventricular wall on DCR images is extremely difficult. Furthermore, the location of the right ventricular wall and left ventricular cavity differs between patients with CTEPH and healthy subjects because of the large differences in pathological conditions.^21^, ^22^, ^23^ However, it is useful to use CT images as a reference for ROI location. Therefore, it is recommended that CTA images be used as a reference for ROI locations where left ventricular motion can be most accurately monitored.

In the present study, we observed that lower PD% values correlated with clinically valuable blood flow images showing higher signal values. This finding implies that PD% could potentially serve as a re‐analysis indicator for evaluating pulmonary blood flow. However, in instances where the right ventricle partially overlaps the left ventricle in frontal views, obtaining precise signal values that accurately reflect left ventricular motion may be challenging. Additionally, patient motion during image acquisition can introduce motion artifacts, potentially affecting signal values on DCR images and complicating the accuracy of measurements. Consequently, under such circumstances, utilizing PD% as a re‐analysis metric may pose challenges.

In relation to these findings, our study had several limitations. Firstly, the area of normal blood flow was determined based on images obtained in the supine position, despite DCR being performed in the standing position. Pulmonary blood flow varies with body position due to gravitational effects, with approximately 20% occurring in the lower lung regions.^24^, ^25^ Therefore, using DCR images acquired in the supine position offers a more accurate and consistent basis for evaluation. Additionally, the evaluation of the colorization effect in pulmonary blood flow images, which visualizes blood flow areas with high correlation coefficients, was conducted solely through visual assessment. Consequently, this study lacked a quantitative assessment of changes in pulmonary blood flow. Future studies should include a quantitative analysis of blood flow signal values using images that visualize the amount of change in the blood flow signal.^14^ Among patients with pulmonary disease, DCR has shown an accuracy of 62.3% in detecting V/Q mismatches compared to lung scintigraphy.^26^ Similarly, in patients with lung cancer, DCR exhibited only a moderate correlation with lung scintigraphy. However, this correlation is significantly influenced by the presence of overlying soft tissue and is diminished in pathological lungs.^27^ These findings collectively suggest that the current methods for quantitatively evaluating pulmonary blood flow signal values using DCR have several notable limitations.

Despite these limitations, the ROI locations that can offer clinically valuable high‐signal‐value DCR‐based pulmonary blood flow images have been identified. Compared to CTA, pulmonary blood flow scintigraphy, and pulmonary angiography, which are currently the mainstream diagnostic imaging modalities for pulmonary blood flow, pulmonary blood flow analysis with DCR is a minimally invasive examination that requires no puncture and uses no RI drugs or contrast medium. Furthermore, the exposure dose was as low as two chest radiographs. In addition, it has an exposure time of 7–10 s with breath‐holding that can be performed with general radiography equipment, making it a promising, low‐cost, and simple method for pulmonary blood flow imaging. Moreover, pulmonary blood flow imaging is useful in diagnosing diseases other than CTEPH. DCR has been shown to correlate with congenital unilateral absence of the pulmonary artery and lung perfusion scintigraphy for the quantitative assessment of pulmonary blood flow laterality.^28^ In addition, DCR correlates well with dual‐energy CT and perfusion scintigraphy in acute pulmonary thromboembolism.^29^ Furthermore, previous studies reported that DCR pixel values correlate with hemodynamics in patients with heart failure, particularly with invasive parameters measured using right heart catheterization.^30^ These findings suggest that DCR is being increasingly used in clinical practice. However, the effect of changes in pulmonary blood flow signal values on diagnostic performance caused by changes in the reference ROI location has not yet been clarified. In this study, the properties of pulmonary blood flow signal values were clarified by changing the reference ROI location and were used to establish the logic for setting the ROI location, which can contribute to low‐cost and simple pulmonary blood flow imaging for CTEPH and various pathological conditions. The ROI locations that provided clinically useful high signal value images obtained in this study are expected to improve the efficiency and reproducibility of the ROI setting, which determines the diagnostic performance of pulmonary blood flow analysis using DCR.

## 5 CONCLUSION

The study identified the ROIs capable of producing high‐signal‐value images in the DCR‐based analysis of pulmonary blood flow, which is crucial for enhancing the efficiency and reliability of pulmonary blood flow imaging in diagnosing CTEPH. This discovery is anticipated to improve the speed and consistency of pulmonary blood flow imaging for CTEPH diagnosis using DCR.

## AUTHOR CONTRIBUTIONS

All authors participated in the data collection, analysis, writing, and editing of the manuscript.

## ETHICS STATEMENT

This retrospective study was approved by the University of Toyama Ethical Review Committee for Clinical and Epidemiological Research (approval number: R2022196), and written informed consent was waived.

## CONFLICT OF INTEREST STATEMENT

The authors declare that they have no conflict of interest.

## Data Availability

The image datasets used in this study are not publicly available due to ethical considerations.
